# From Awareness to Action: Bridging Knowledge and Practice Gaps in Contraceptive Counselling for Women with Chronic Medical Conditions—A Cross-Sectional Survey

**DOI:** 10.3390/healthcare14172864

**Published:** 2026-09-05

**Authors:** Eda Güner Özen, Süleyman Özen, Ahkam Göksel Kanmaz, Yaşam Kemal Akpak

**Affiliations:** 1 Department of Obstetrics and Gynecology, İzmir City Hospital, İzmir 35040, Türkiye; 2 Department of Gynecologic Oncology, İzmir City Hospital, İzmir 35040, Türkiye; 3 Department of Obstetrics and Gynecology, İzmir Faculty of Medicine, University of Health Sciences, İzmir 35040, Türkiye

**Keywords:** chronic disease, contraception, reproductive health, contraceptive counselling, WHO medical eligibility criteria

## Abstract

**Highlights:**

**What are the main findings?**

- Most physicians recognized the importance of contraception, but self-reported routine contraceptive counselling remained limited.
- WHO MEC awareness and postgraduate contraception training remained associated with higher odds of self-reported routine counselling after adjustment for potential confounders.

**What are the implications of the main findings?**

- Contraceptive counselling should be more systematically integrated into chronic disease management across non-obstetric specialties.
- Broader dissemination of the WHO MEC, continuing professional education, and organizational support may facilitate the integration of contraceptive counselling into routine care.

**Abstract:**

**Background:** Women with chronic medical conditions are at increased risk of adverse pregnancy outcomes, making contraceptive counselling an essential component of comprehensive chronic disease management. However, evidence regarding the knowledge and clinical practices of non-obstetric physicians remains limited. This study evaluated physicians’ knowledge, attitudes, and practices regarding contraceptive counselling for women with chronic medical conditions in a tertiary referral hospital in Türkiye. **Methods:** A cross-sectional Knowledge–Attitude–Practice survey was conducted among 264 non-obstetric physicians at İzmir City Hospital between July and October 2025. The questionnaire assessed contraceptive counselling practices, awareness of the World Health Organization Medical Eligibility Criteria for Contraceptive Use (WHO MEC), postgraduate contraception training, perceived barriers, and educational needs. Categorical associations were examined using chi-square or exact procedures as appropriate, and multivariable binary logistic regression was performed to evaluate factors associated with self-reported routine contraceptive counselling while accounting for potential confounding. **Results:** Among the 260 physicians who responded to the relevant item, 89.2% agreed that contraceptive method selection may influence the course of chronic disease, whereas 37.9% of the total sample reported routinely providing contraceptive counselling. Awareness of the WHO MEC was limited, with 75.8% reporting no prior awareness, and 18.2% had received postgraduate contraception training. In the adjusted analysis, WHO MEC awareness (aOR = 2.76, 95% CI: 1.39–5.46; *p* = 0.004), postgraduate contraception training (aOR = 2.38, 95% CI: 1.12–5.07; *p* = 0.024), seeing ≥30 women of reproductive age per week (aOR = 2.07, 95% CI: 1.15–3.73; *p* = 0.016), female sex (aOR = 3.81, 95% CI: 1.90–7.65; *p* < 0.001), and >10 years of clinical practice (aOR = 2.38, 95% CI: 1.23–4.62; *p* = 0.010) were associated with higher odds of self-reported routine counselling. Specialty was not significantly associated after adjustment. **Conclusions:** Self-reported routine contraceptive counselling remained limited despite broad recognition of the relevance of contraception in chronic disease care. Guideline awareness, postgraduate training, clinical exposure, sex, and clinical experience were associated with routine counselling after adjustment, although causal relationships cannot be inferred from this cross-sectional study. These findings support further evaluation of educational and organizational strategies for integrating reproductive healthcare into chronic disease management.

## 1. Introduction

Women with chronic medical conditions represent a growing and increasingly vulnerable subgroup within the reproductive-age population. Advances in medical care have improved survival and long-term disease control in cardiovascular, renal, autoimmune, and metabolic disorders, enabling more women with chronic illnesses to reach childbearing age. This demographic shift reflects the global increase in non-communicable diseases and disability burden documented in recent epidemiological analyses [1]. Consequently, reproductive health assessment and pregnancy planning have become increasingly important components of comprehensive chronic disease management.

Maternal mortality remains a key indicator of health system performance. According to the World Health Organization (WHO), a substantial proportion of maternal deaths are preventable through timely access to high-quality reproductive healthcare and appropriate preconception risk management [2]. Although direct obstetric complications account for most maternal deaths worldwide, indirect causes, particularly those related to pre-existing chronic medical conditions, also contribute substantially to preventable maternal mortality [2]. In Türkiye, the maternal mortality ratio has been reported as 29 per 100,000 live births [3]. A 20-year regional analysis further demonstrated that 39.2% of maternal deaths were attributable to indirect causes, including cardiovascular and other chronic diseases [4]. These findings underscore the importance of integrating reproductive risk assessment and preventive strategies into the care of women with chronic illnesses before conception.

Unintended pregnancy in women with chronic medical conditions presents a substantial clinical challenge. Previous population-based studies have also shown that the use of highly effective contraception among women with chronic diseases remains suboptimal despite their increased reproductive risks [5]. Pregnancy during periods of disease instability or exposure to potentially teratogenic medications may increase maternal and perinatal morbidity [6]. Contraceptive counselling should therefore be regarded as an essential component of female reproductive healthcare and preconception care rather than as a secondary aspect of chronic disease management [6]. The national reproductive health context further underscores the relevance of this issue in Türkiye. According to the 2018 Turkey Demographic and Health Survey (TDHS), 70% of currently married women aged 15–49 years were using a contraceptive method, while 49% were using a modern method [7]. In the same survey, 11% of recent births and ongoing pregnancies were reported as mistimed and 15% as unwanted, indicating that approximately 26% were unintended [7]. Despite this need, family planning practices in Türkiye remain variable and are influenced, in part, by healthcare providers’ knowledge and clinical priorities [8]. Modern contraceptive use also remains suboptimal, and counselling practices are inconsistent across healthcare settings [9].

The World Health Organization Medical Eligibility Criteria for Contraceptive Use (WHO MEC) provides an evidence-based framework for selecting safe and appropriate contraceptive methods for women with specific medical conditions [10]. However, evidence regarding the recognition and application of this guidance by non-obstetric physicians remains limited. In a small physician-based survey conducted at a tertiary referral hospital in India, contraceptive history was frequently overlooked, counselling and referral practices were inconsistent, and only a minority of physicians were able to identify the WHO MEC correctly [11]. The authors also emphasized that contraceptive care for women with medical disorders should not be regarded as the exclusive responsibility of obstetricians and gynaecologists [11]. These findings suggest that important gaps may persist among specialists who routinely manage chronic diseases, particularly in tertiary referral hospitals where women with medically complex conditions are treated and potentially teratogenic therapies are prescribed.

Therefore, this study aimed to evaluate the knowledge, attitudes, and self-reported practices of non-obstetric physicians regarding contraceptive counselling for women of reproductive age with chronic medical conditions in a tertiary referral hospital in Türkiye. The study further sought to identify provider- and system-level gaps that may inform future educational initiatives and improvements in the integration of reproductive healthcare into multidisciplinary chronic disease management.

## 2. Methods

### 2.1. Study Design and Setting

This single-center, cross-sectional Knowledge–Attitude–Practice (KAP) survey was conducted among non-obstetrician–gynaecologist physicians at İzmir City Hospital, a tertiary referral hospital in Türkiye, between July and October 2025. The study was designed to evaluate physicians’ knowledge, attitudes, and self-reported practices regarding contraceptive counselling for women of reproductive age with chronic medical conditions.

### 2.2. Participants

Eligible participants were physicians from non-obstetric and non-gynaecological medical and surgical specialties who routinely provided care to women of reproductive age. Participants were recruited using convenience sampling. An electronic survey link was shared with potentially eligible physicians through the institutional multidisciplinary meeting group, and participation was voluntary and anonymous. Because the total number of eligible physicians who received or had access to the survey link was not prospectively recorded, the number of physicians invited to participate and, consequently, a reliable response rate could not be determined.

A total of 264 physicians with analysable responses were included in the study. Physicians who did not routinely manage women of reproductive age were not eligible to participate.

### 2.3. Questionnaire Development

The questionnaire was developed by adapting items from previously published physician-based surveys addressing contraceptive knowledge, attitudes, and counselling practices [11,12]. The investigators reviewed the draft questionnaire to ensure content relevance, clinical applicability, and clarity in the context of non-obstetric physicians caring for women with chronic medical conditions.

The final questionnaire consisted of 27 items. Items 1–5 assessed demographic and professional characteristics; Items 6–12 addressed clinical and contraceptive counselling practices; Items 13–18 assessed knowledge, attitudes, and perceived barriers; Items 19–23 addressed postgraduate education and self-perceived competence; Items 24–26 assessed service-related factors and sources of information; and Item 27 elicited suggestions for improving contraceptive counselling services. The questionnaire included single-choice, Likert-type, and multiple-response items, depending on the construct being assessed. The complete questionnaire is provided in Supplementary File S1.

The draft questionnaire was pilot-tested in 10 physicians to assess face validity, comprehensibility, and clinical relevance. Minor wording and clarity revisions were made following pilot testing, without substantive changes to the questionnaire content or response structure. Pilot participants were not included in the final study sample.

The questionnaire was designed to assess several conceptually distinct aspects of knowledge, attitudes, practices, barriers, educational needs, and service-related factors rather than to generate a single composite KAP score. Accordingly, an overall Cronbach’s alpha was not considered appropriate. However, no formal psychometric validation or domain-specific reliability analysis was performed. The questionnaire should therefore be regarded as an adapted survey instrument with investigator review and pilot-based face validity rather than as a formally validated psychometric scale.

### 2.4. Statistical Analysis

Data were analysed using IBM SPSS Statistics version 25.0 (IBM Corp., Armonk, NY, USA). Descriptive statistics are presented as frequencies and percentages. Associations between categorical variables were evaluated using Pearson’s χ^2^ test when expected cell frequencies were adequate. When expected cell frequencies were sparse, Fisher’s exact test was used for 2 × 2 contingency tables, whereas a Monte Carlo fixed-margin exact chi-square procedure based on 200,000 simulated tables was used for larger contingency tables. All tests were two-sided, and *p* values < 0.05 were considered statistically significant. For multiple-response items, each response option was analysed as a separate binary variable (selected versus not selected).

Self-reported routine provision of contraceptive counselling was dichotomised as “Yes” for responses of “Agree” or “Strongly agree” and “No” for responses of “Neutral,” “Disagree,” or “Strongly disagree.” Bivariable associations with weekly clinical exposure, WHO MEC awareness, postgraduate contraception training, sex, specialty, and years of clinical practice were initially examined, followed by multivariable analysis to account for potential confounding.

A multivariable binary logistic regression model was fitted with self-reported routine contraceptive counselling as the dependent variable. The model simultaneously included WHO MEC awareness (any awareness vs. never heard), postgraduate contraception training (any training vs. none), weekly clinical exposure (≥30 vs. <30 women of reproductive age per week), sex (female vs. male), specialty (medical vs. surgical), and years of clinical practice (>10 vs. ≤10 years). Age was not included simultaneously with years of clinical practice because of the strong correlation between these variables (phi = 0.747; Spearman *r* = 0.707). Results are reported as adjusted odds ratios (aORs) with 95% confidence intervals (CIs) and two-sided *p* values.

Missingness was assessed at the item level. No missing responses were identified for variables included in Table 1, Table 2, Table 3 and Table 4 or in the multivariable regression analysis (N = 264). For supplementary questionnaire items with missing responses, percentages were calculated using the number of available responses for the relevant item rather than the total study sample; the applicable denominator is reported in Supplementary Table S1. No imputation of missing data was performed.

Model performance and diagnostics were assessed using the likelihood-ratio test, the area under the receiver operating characteristic curve (AUC), the Hosmer–Lemeshow goodness-of-fit test, and variance inflation factors (VIFs) for multicollinearity. A bootstrap 95% confidence interval was calculated for the AUC. Because the model was intended to estimate adjusted associations rather than to serve as a clinical prediction model, these measures were used to assess model performance and adequacy rather than predictive validity.

Statistical analyses were conducted with the support of an independent professional biostatistics consultancy service. The authors retained full responsibility for data interpretation and reporting.

### 2.5. Ethics Approval and Consent to Participate

The study was conducted in accordance with the Declaration of Helsinki and approved by the İzmir City Hospital Non-Interventional Research Ethics Committee (protocol code 2025/268; date of approval: 4 June 2025). Electronic informed consent was obtained from all participants before participation. Participants could proceed to the survey only after providing consent. All data were collected anonymously. 

## 3. Results

### 3.1. Participant Characteristics

A total of 264 physicians were included in the analysis (Table 1). Of these, 68.2% were female, and 81.8% were between 30 and 39 years of age. Medical specialties accounted for 72.7% of participants, while 27.3% practiced in surgical specialties.

Regarding clinical experience, 60.6% of participants had 6–10 years of practice, and 24.2% had more than 10 years of clinical experience. Overall, 47.0% reported seeing ≥30 women of reproductive age per week. Comparisons according to age group, years of clinical practice, and specialty are presented in Table 2. Comparisons according to weekly clinical exposure are presented in Table 3.

### 3.2. Clinical Practices in Contraceptive Counselling

Responses related to contraceptive counselling practices are presented in Supplementary Table S1. During the preceding three months, 62.1% of physicians reported providing no pregnancy planning or contraceptive counselling to women with chronic medical conditions, whereas 12.1% reported counselling seven or more patients.

The distribution of self-reported counselling frequency differed according to age group and years of clinical practice (Table 2; *p* = 0.002 and *p* = 0.016, respectively). Physicians aged ≤39 years and those with ≤10 years of clinical practice more frequently reported counselling seven or more patients during the preceding three months than physicians aged ≥40 years and those with >10 years of clinical practice.

Regarding contraceptive methods, 51.5% of participants reported feeling competent to recommend combined oral contraceptives and 34.8% reported competence in recommending barrier methods. A total of 21.2% reported not feeling competent to recommend any contraceptive method. The proportion of physicians reporting competence in recommending combined oral contraceptives differed between medical and surgical specialties (58.3% vs. 33.3%, *p* < 0.001) (Table 2).

Overall, 81.8% of participants agreed or strongly agreed that they checked contraceptive use before prescribing potentially teratogenic medications, while 57.6% agreed or strongly agreed that they evaluated potential drug–contraceptive interactions. Reassessment of combined hormonal contraceptive use during prolonged immobilisation was reported by 66.7% of participants. Overall, 37.9% of participants agreed or strongly agreed that they routinely provided contraceptive counselling to women with chronic medical conditions (Supplementary Table S1).

### 3.3. Knowledge, Training, and Perceived Barriers

Responses related to knowledge, training, and perceived barriers are summarised in Supplementary Table S1. Among the 260 physicians who responded to this item, 89.2% agreed that contraceptive method selection may influence the course of chronic disease.

Most participants (75.8%) reported no prior awareness of the World Health Organization Medical Eligibility Criteria for Contraceptive Use (WHO MEC), whereas 3.0% reported detailed familiarity. WHO MEC awareness differed according to age group (*p* = 0.006) and weekly clinical exposure (*p* < 0.001), but not according to years of clinical practice (*p* = 0.229) or specialty (*p* = 0.345) (Table 2 and Table 3).

Formal postgraduate training in contraception was reported by 18.2% of participants. Training differed according to age group and years of clinical practice (both *p* < 0.001), but not according to specialty (*p* = 0.946). The proportion of physicians reporting postgraduate contraception training did not differ significantly according to weekly clinical exposure (*p* = 0.498) (Table 3).

Self-rated knowledge of contraception is presented in Supplementary Table S1. No participant rated their knowledge as “very good.” Reported barriers to contraceptive counselling included perceiving the topic as outside one’s specialty (50.0%), lack of up-to-date knowledge (48.5%), and limited consultation time (47.0%). Overall, 65.2% expressed willingness to participate in an online educational programme, and video-based training was the preferred educational format (Supplementary Table S1).

### 3.4. Associations Between Weekly Clinical Exposure and Counselling Practices

Comparisons according to weekly clinical exposure are presented in Table 3. The distribution of self-reported routine contraceptive counselling differed significantly across weekly clinical exposure groups (*p* < 0.001), as did the number of patients counselled during the preceding three months (*p* < 0.001). WHO MEC awareness also differed significantly according to weekly clinical exposure (*p* < 0.001). In contrast, postgraduate contraception training did not differ significantly across weekly clinical exposure groups (*p* = 0.498).

### 3.5. Factors Associated with Self-Reported Routine Contraceptive Counselling

Multivariable binary logistic regression analysis was performed to evaluate factors associated with self-reported routine contraceptive counselling while accounting for potential confounding (Table 4). WHO MEC awareness was associated with higher odds of self-reported routine contraceptive counselling (aOR = 2.76, 95% CI: 1.39–5.46; *p* = 0.004). Postgraduate contraception training was also associated with higher odds of counselling (aOR = 2.38, 95% CI: 1.12–5.07; *p* = 0.024). In addition, physicians who reported seeing ≥30 women of reproductive age per week had higher odds of reporting routine contraceptive counselling (aOR = 2.07, 95% CI: 1.15–3.73; *p* = 0.016). Female physicians (aOR = 3.81, 95% CI: 1.90–7.65; *p* < 0.001) and physicians with >10 years of clinical practice (aOR = 2.38, 95% CI: 1.23–4.62; *p* = 0.010) likewise had higher odds of reporting routine counselling. Medical versus surgical specialty was not significantly associated with self-reported routine contraceptive counselling after adjustment (aOR = 1.23, 95% CI: 0.65–2.34; *p* = 0.522).

The overall model was statistically significant (likelihood-ratio χ^2^ = 53.20, df = 6, *p* < 0.001) and showed acceptable discrimination (AUC = 0.759; bootstrap 95% CI: 0.700–0.816). Multicollinearity was low (maximum VIF = 1.26). However, the Hosmer–Lemeshow goodness-of-fit test was significant (χ^2^ = 22.91, df = 7, *p* = 0.0018), indicating imperfect calibration. Accordingly, the adjusted estimates were interpreted cautiously as measures of association rather than as evidence of predictive performance.

## 4. Discussion

In this single-center cross-sectional study of non-obstetrician–gynaecologist physicians, self-reported routine contraceptive counselling remained limited despite frequent clinical contact with women of reproductive age and broad recognition of the importance of contraception in chronic disease care. In the multivariable analysis, WHO MEC awareness, postgraduate contraception training, seeing ≥30 women of reproductive age per week, female sex, and >10 years of clinical practice were associated with higher odds of self-reported routine contraceptive counselling, whereas medical versus surgical specialty was not significantly associated after adjustment. These findings suggest that the incorporation of contraceptive counselling into routine care may reflect a combination of guideline familiarity, educational exposure, clinical experience, and physician characteristics rather than specialty affiliation alone. Given the cross-sectional design, however, these associations should not be interpreted as causal relationships.

The discrepancy between recognition of the importance of contraception and its routine incorporation into clinical care remains an important finding. Similar patterns have been reported in physician-based studies conducted in different healthcare settings. Prasad et al. [11] found that physicians caring for women with chronic diseases generally recognized the importance of contraception but demonstrated deficiencies in contraceptive knowledge and counselling practices. Likewise, Miranda-Silva et al. [12] observed variability in physicians’ contraceptive counselling practices and limited familiarity with evidence-based contraceptive recommendations. Together with our findings, these studies indicate that recognition of reproductive health needs does not necessarily translate into consistent self-reported counselling practice. Rather than repeatedly framing this discrepancy as an awareness–implementation gap, our findings point to specific potentially modifiable factors—particularly guideline familiarity and postgraduate education—that may be relevant to improving counselling delivery.

This issue is clinically important because women with chronic medical conditions frequently receive long-term care from physicians outside obstetrics and gynaecology. Non-obstetric physicians may therefore have repeated opportunities to discuss pregnancy intentions, reproductive planning, and contraceptive options. International recommendations increasingly emphasize integrating reproductive healthcare into chronic disease management rather than restricting it to specialist reproductive health services [6,10]. The WHO Medical Eligibility Criteria were developed to facilitate evidence-based contraceptive decision-making across a wide spectrum of medical conditions [9,10], while the American College of Obstetricians and Gynecologists emphasizes opportunities to discuss reproductive goals and optimize chronic disease management before pregnancy [6]. The relatively low prevalence of routine self-reported counselling in our study suggests that these principles may not yet be consistently incorporated into clinical practice within the study setting.

Limited WHO MEC awareness was another notable finding. Approximately three-quarters of participating physicians reported no prior awareness of the guideline, and only a small minority reported detailed familiarity. After adjustment for other physician and clinical characteristics, any WHO MEC awareness was associated with higher odds of self-reported routine contraceptive counselling (aOR = 2.76, 95% CI: 1.39–5.46). Comparable deficiencies in familiarity with contraceptive eligibility recommendations have been reported internationally [11,12]. The WHO developed the MEC to support safe contraceptive decision-making for individuals with a range of medical conditions and to reduce unnecessary medical barriers to contraception [10]. Experienced primary care physicians have also described the US MEC as a useful point-of-care resource for contraceptive decision-making in patients with medical conditions [13]. Although our cross-sectional findings cannot establish that MEC awareness increases counselling behaviour, the adjusted association supports the relevance of improving access to and familiarity with evidence-based contraceptive guidance among non-obstetric physicians.

Postgraduate contraception training was similarly uncommon. Nevertheless, physicians reporting any postgraduate contraception training had higher adjusted odds of reporting routine contraceptive counselling (aOR = 2.38, 95% CI: 1.12–5.07). Previous studies have identified inadequate formal education as an important barrier to physicians’ confidence and involvement in contraceptive counselling [11,12]. The WHO has also emphasized that publication of evidence-based recommendations alone may be insufficient to change practice without dissemination, professional education, and implementation within healthcare systems [10]. Our findings therefore support postgraduate education as a potentially modifiable factor associated with counselling practice, while the observational design prevents determining whether training itself produced the observed difference.

Clinical exposure was also associated with counselling after adjustment. Physicians seeing ≥30 women of reproductive age per week had higher odds of reporting routine contraceptive counselling than those with lower weekly exposure (aOR = 2.07, 95% CI: 1.15–3.73). Greater exposure may increase opportunities to encounter reproductive health concerns and reinforce the perceived relevance of contraceptive counselling. Conversely, physicians already interested or experienced in women’s health may work in settings with greater exposure to reproductive-aged women. Because temporality cannot be established, both explanations remain possible. Notably, postgraduate contraception training did not differ significantly across weekly clinical exposure groups in the revised analysis, indicating that greater clinical exposure should not be interpreted as a proxy for formal training. Previous physician-based studies and WHO implementation guidance similarly support accessible educational resources and practical contraceptive guidance across clinical settings [10,11,12].

The findings regarding age and clinical experience require particular consideration. In the categorical analyses, physicians aged ≤39 years and those with ≤10 years of clinical practice more frequently reported counselling seven or more patients during the preceding three months than their respective older or more experienced comparison groups. In contrast, >10 years of clinical practice was associated with higher odds of self-reported routine counselling in the multivariable model (aOR = 2.38, 95% CI: 1.23–4.62). These findings are not necessarily contradictory because the analyses assessed related but distinct outcomes: the number of patients counselled during a defined three-month period and physicians’ self-reported routine provision of counselling. Greater recent counselling volume among younger physicians could reflect differences in clinical workload, patient mix, or service roles, whereas greater clinical experience may influence whether counselling is perceived as an established component of routine practice. These potential mechanisms were not measured in the present study and should therefore be regarded as hypotheses rather than explanations established by the data. Age was not entered simultaneously with years of clinical practice in the final multivariable model because of the strong correlation between these variables.

Female physicians also had higher adjusted odds of reporting routine contraceptive counselling than male physicians (aOR = 3.81, 95% CI: 1.90–7.65). The study was not designed to determine the mechanisms underlying this association, and potentially relevant factors such as differences in patient communication, case mix, consultation patterns, or patient preferences were not assessed. This finding should therefore be interpreted cautiously and warrants further investigation. Conversely, medical versus surgical specialty was not significantly associated with routine counselling after adjustment. This finding suggests that counselling behaviour may not be explained by broad specialty classification alone and supports considering reproductive counselling needs across non-obstetric disciplines.

The clinical relevance of contraceptive counselling extends beyond physician knowledge. Women with chronic medical conditions may face increased risks when pregnancy occurs in the context of poorly controlled disease or exposure to potentially teratogenic medications. Reproductive counselling has therefore been emphasized in clinical contexts such as anticoagulation therapy, where safe prescribing requires consideration of pregnancy intentions and contraceptive needs [14]. Hunter-Greaves et al. [15] reported that women with chronic diseases may receive insufficient disease-specific contraceptive counselling and consequently use methods that are not optimally aligned with their underlying conditions. Similarly, the systematic review and meta-analysis by Feyissa et al. [16] identified substantial variability in contraceptive counselling among women with cardiovascular disease and persistent deficiencies in disease-specific reproductive counselling. These observations reinforce the clinical relevance of integrating reproductive considerations into chronic disease management, while our data specifically demonstrate gaps in physicians’ self-reported counselling rather than directly measured patient-level care or outcomes.

The findings also have practical implications for intervention development. Participants reported several barriers, including insufficient up-to-date knowledge, limited consultation time, and the perception that contraceptive counselling falls outside their specialty. Similar barriers have been reported in other physician-based studies [11,12]. Accordingly, education alone may be insufficient. Potential organizational approaches include standardized assessment of reproductive intentions, readily accessible decision-support tools based on current recommendations, structured referral pathways, and prompts when potentially teratogenic medications are prescribed [10,14]. These strategies were not evaluated in the present study and should therefore be regarded as potential interventions for future evaluation rather than interventions demonstrated to be effective by our data. Point-of-care use of contraceptive eligibility guidance may also facilitate evidence-based decision-making during routine consultations [13].

The high level of interest in online education among participants provides an additional opportunity for future intervention. Digital educational modules, case-based learning, and accessible clinical decision-support resources could potentially facilitate continuing professional education without substantially disrupting clinical workflows. Patient decision aids may also improve contraceptive knowledge and facilitate shared decision-making, although evidence regarding longer-term behavioural outcomes remains limited [17]. Future studies should evaluate whether combining clinician education with workflow-based interventions produces measurable improvements in observed counselling behaviour and patient-level reproductive health outcomes.

Although conducted among obstetricians and gynaecologists, a Turkish survey also demonstrated variability in contraceptive counselling practices and preferences, suggesting that continuing professional education may remain relevant even among clinicians with reproductive healthcare expertise [18]. Together with the present findings, this supports viewing contraceptive counselling as a multidisciplinary healthcare-delivery issue rather than one determined solely by specialty. However, because our participants were recruited from a single tertiary-care institution, the present findings should not be interpreted as representative of physicians throughout Türkiye.

This study has several strengths. It evaluated contraceptive counselling across multiple non-obstetric medical and surgical specialties and simultaneously examined WHO MEC awareness, postgraduate contraception training, perceived barriers, clinical exposure, and practices related to potentially teratogenic medications. The revised multivariable analysis further allowed the associations with self-reported routine counselling to be evaluated while accounting for several potential confounding factors.

Several limitations should also be considered. First, the cross-sectional design prevents establishing temporal or causal relationships between physician characteristics, education, guideline awareness, and counselling practices. Second, the study used convenience sampling at a single tertiary-care institution, limiting external validity; the findings therefore cannot be assumed to represent non-obstetrician–gynaecologist physicians in Türkiye as a whole. In addition, the survey link was distributed electronically to potentially eligible physicians during the institutional recruitment process, but the total number of physicians who received or had access to the link was not prospectively recorded. Consequently, a reliable response rate could not be calculated, and participation bias cannot be excluded. Third, counselling practices were self-reported rather than directly observed and may therefore be affected by recall and social-desirability bias; statements regarding counselling in this study refer to physicians’ reported practices and should not be interpreted as direct measures of actual clinical behaviour. Fourth, although the questionnaire was adapted from previously published instruments and underwent investigator review and pilot testing for face validity, it did not undergo formal psychometric validation. This limits conclusions regarding the measurement properties of the questionnaire and should be considered when interpreting domain-specific responses. Fifth, although the multivariable model showed acceptable discrimination and low multicollinearity, the significant Hosmer–Lemeshow test indicated imperfect calibration. The adjusted estimates should therefore be interpreted cautiously as exploratory associations rather than as evidence of predictive performance. Finally, although multivariable regression was used to account for measured potential confounders, residual confounding by unmeasured physician-, patient-, or institution-level factors remains possible.

## 5. Conclusions

In this single-center cross-sectional study, non-obstetrician–gynaecologist physicians generally recognized the importance of contraception in the care of women with chronic medical conditions, yet self-reported routine contraceptive counselling remained limited. In the adjusted analysis, WHO MEC awareness, postgraduate contraception training, higher weekly clinical exposure to women of reproductive age, female sex, and >10 years of clinical practice were associated with higher odds of self-reported routine counselling, whereas broad specialty classification was not significantly associated. These associations should not be interpreted as causal because of the cross-sectional design. The findings support the potential value of improving access to evidence-based contraceptive guidance and continuing professional education while also addressing organizational barriers to integrating reproductive health discussions into chronic disease care. Given the single-center convenience sample and reliance on self-reported practices, the findings should not be generalized to physicians throughout Türkiye without confirmation in larger, multicenter studies. Future research should evaluate whether educational and workflow-based interventions translate into objectively measured improvements in contraceptive counselling and patient-level reproductive health outcomes.

## Figures and Tables

**Table 1:** Demographic and Professional Characteristics of Participants (N = 264).

Variable	Category	*n* (%)
**Sex**	Female	180 (68.2)
	Male	84 (31.8)
**Age group (years)**	<30	8 (3.0)
	30–39	216 (81.8)
	40–49	28 (10.6)
	≥50	12 (4.5)
**Specialty**	Medical specialties	192 (72.7)
	Surgical specialties	72 (27.3)
**Years in clinical practice**	0–5 years	40 (15.2)
	6–10 years	160 (60.6)
	11–15 years	28 (10.6)
	16–20 years	16 (6.1)
	≥21 years	20 (7.6)
**Weekly number of women of reproductive age seen**	<10	64 (24.2)
	10–20	52 (19.7)
	21–30	24 (9.1)
	≥30	124 (47.0)

**Legend:** Data are presented as number (percentage). The bold formatting is used to distinguish the variable headings from their categories and has been retained consistently throughout the table.

**Table 2:** Physician Age, Clinical Experience, and Specialty in Relation to Contraceptive Counselling Knowledge and Practice (N = 264).

Variable	Category	≤39 yrs *n* (%)	≥40 yrs *n* (%)	*p*	≤10 yrs *n* (%)	>10 yrs *n* (%)	*p*	Medical *n* (%)	Surgical *n* (%)	*p*
Counselling frequency (past 3 months)	None	140 (62.5)	24 (60.0)	0.002 †	124 (62.0)	40 (62.5)	0.016 †	—	—	—
	1–3	40 (17.9)	16 (40.0)		36 (18.0)	20 (31.3)		—	—	
	4–6	12 (5.4)	0 (0)		12 (6.0)	0 (0)		—	—	
	≥7	32 (14.3)	0 (0)		28 (14.0)	4 (6.3)		—	—	
Provision of counselling (Likert)	Strongly agree	40 (17.9)	4 (10.0)	<0.001	32 (16.0)	12 (18.8)	<0.001	36 (18.8)	8 (11.1)	<0.001
	Agree	44 (19.6)	12 (30.0)		36 (18.0)	20 (31.3)		40 (20.8)	16 (22.2)	
	Neutral	32 (14.3)	20 (50.0)		32 (16.0)	20 (31.3)		28 (14.6)	24 (33.3)	
	Disagree	60 (26.8)	4 (10.0)		56 (28.0)	8 (12.5)		56 (29.2)	8 (11.1)	
	Strongly disagree	48 (21.4)	0 (0)		44 (22.0)	4 (6.3)		32 (16.7)	16 (22.2)	
WHO MEC awareness	Never heard	176 (78.6)	24 (60.0)	0.006 †	152 (76.0)	48 (75.0)	0.229 †	144 (75.0)	56 (77.8)	0.345 †
	Heard but not familiar	40 (17.9)	16 (40.0)		40 (20.0)	16 (25.0)		40 (20.8)	16 (22.2)	
	Know in detail	8 (3.6)	0 (0)		8 (4.0)	0 (0)		8 (4.2)	0 (0)	
Postgraduate training	None	196 (87.5)	20 (50.0)	<0.001	172 (86.0)	44 (68.8)	<0.001	156 (81.3)	60 (83.3)	0.946
	Any training	28 (12.5)	20 (50.0)		28 (14.0)	20 (31.3)		36 (18.8)	12 (16.7)	
Self-perceived competence in COC	Yes	—	—	—	—	—	—	112 (58.3)	24 (33.3)	<0.001
	No	—	—	—	—	—	—	80 (41.7)	48 (66.7)	

**Legend:** Data are presented as *n* (%). Percentages are calculated within the corresponding age, clinical experience, or specialty group. Pearson’s χ^2^ test was used when expected cell frequencies were adequate. For contingency tables with sparse expected cell frequencies, a Monte Carlo fixed-margin exact chi-square procedure based on 200,000 simulated tables was used. All tests were two-sided, and *p* values < 0.05 were considered statistically significant. † Monte Carlo exact *p* value. WHO MEC, World Health Organization Medical Eligibility Criteria; COC, combined oral contraceptive.

**Table 3:** Weekly Clinical Exposure to Women of Reproductive Age in Relation to Contraceptive Counselling Knowledge and Practice (N = 264).

Variable	Category	<10 *n* (%)	10–20 *n* (%)	21–30 *n* (%)	≥30 *n* (%)	*p*
**Provision of Counselling (Likert)**	Strongly agree	4 (6.2)	4 (7.7)	4 (16.7)	32 (25.8)	<0.001 †
	Agree	16 (25.0)	8 (15.4)	8 (33.3)	24 (19.4)	
	Neutral	24 (37.5)	8 (15.4)	4 (16.7)	16 (12.9)	
	Disagree	8 (12.5)	16 (30.8)	0 (0.0)	40 (32.3)	
	Strongly disagree	12 (18.8)	16 (30.8)	8 (33.3)	12 (9.7)	
**Counselling in Past 3 Months**	None	64 (100.0)	40 (76.9)	16 (66.7)	44 (35.5)	<0.001 †
	1–3	0 (0.0)	8 (15.4)	4 (16.7)	44 (35.5)	
	4–6	0 (0.0)	0 (0.0)	4 (16.7)	8 (6.5)	
	≥7	0 (0.0)	4 (7.7)	0 (0.0)	28 (22.6)	
**WHO MEC Awareness**	Never heard	60 (93.8)	44 (84.6)	16 (66.7)	80 (64.5)	<0.001 †
	Heard but not familiar	4 (6.3)	8 (15.4)	8 (33.3)	36 (29.0)	
	Know in detail	0 (0.0)	0 (0.0)	0 (0.0)	8 (6.5)	
**Postgraduate Training**	None	52 (81.3)	44 (84.6)	20 (83.3)	100 (80.6)	0.498 †
	Any training	12 (18.8)	8 (15.4)	4 (16.7)	24 (19.4)	

**Legend:** Data are presented as *n* (%), with percentages calculated within each weekly clinical exposure group. Because of sparse expected cell frequencies, comparisons were performed using a Monte Carlo fixed-margin exact chi-square procedure based on 200,000 simulated tables. All tests were two-sided, and *p* values < 0.05 were considered statistically significant. † Monte Carlo exact *p* value. WHO MEC, World Health Organization Medical Eligibility Criteria.

**Table 4:** Multivariable Logistic Regression Analysis of Factors Associated with Self-Reported Routine Contraceptive Counselling (N = 264).

Variable	Comparison	aOR	95% CI	*p*
WHO MEC awareness	Any awareness vs. never heard	2.76	1.39–5.46	0.004
Postgraduate contraception training	Any training vs. none	2.38	1.12–5.07	0.024
Weekly clinical exposure	≥30 vs. <30 women/week	2.07	1.15–3.73	0.016
Sex	Female vs. male	3.81	1.90–7.65	<0.001
Specialty	Medical vs. surgical	1.23	0.65–2.34	0.522
Years in clinical practice	>10 vs. ≤10 years	2.38	1.23–4.62	0.010

**Legend:** The dependent variable was self-reported routine contraceptive counselling, dichotomised as “Yes” for responses of “Agree” or “Strongly agree” and “No” for responses of “Neutral,” “Disagree,” or “Strongly disagree.” All variables shown were entered simultaneously into the multivariable binary logistic regression model. Age was not included simultaneously with years of clinical practice because of the strong correlation between these variables (phi = 0.747; Spearman *r* = 0.707). Results are presented as adjusted odds ratios (aORs) with 95% confidence intervals (CIs). Two-sided *p* values < 0.05 were considered statistically significant. WHO MEC, World Health Organization Medical Eligibility Criteria.

## Data Availability

The datasets generated and analyzed during the current study are available from the corresponding author upon reasonable request due to privacy considerations.

## Supplementary Materials

The following supporting information can be downloaded at: https://www.mdpi.com/article/10.3390/healthcare14172864/s1, Supplementary File S1: Questionnaire assessing physicians’ knowledge, attitudes, and practices regarding contraceptive counselling for women with chronic medical conditions. Supplementary Table S1: Clinical Practices, Knowledge, Training, Perceived Barriers, and System-Level Factors Related to Contraceptive Counselling (N = 264).

## Abbreviations

KAP	Knowledge–Attitude–Practice
LARC	long-acting reversible contraception
MEC	Medical Eligibility Criteria
WHO	World Health Organization
non-OB/GYN	non-obstetrics and gynecology
