# Supplementary material for: From Awareness to Action: Bridging Knowledge and Practice Gaps in Contraceptive Counselling for Women with Chronic Medical Conditions—A Cross-Sectional Survey
Source: Healthcare (Basel). 2026 Sep 5;14(17):2864. doi: 10.3390/healthcare14172864 (PMC13566685; doi:10.3390/healthcare14172864)
Supplement: Supplementary file 1 [file healthcare-14-02864-s001.zip › healthcare-4493133-supplementary.pdf]

Supplementary File S1

Supplementary Table S1. Clinical Practices, Knowledge, Training, Perceived Barriers, and System-Level Factors Related to Contraceptive Counselling (N = 264)

Domain A. Clinical Practices and Counselling Behaviour

Variable	Category	n (%)
**Contraceptive counselling activity (past 3 months)**	None	164 (62.1)
	1–3 patients	56 (21.2)
	4–6 patients	12 (4.5)
	≥7 patients	32 (12.1)
**Self-reported routine counselling behaviour**	Strongly agree	44 (16.7)
	Agree	56 (21.2)
	Neutral	52 (19.7)
	Disagree	64 (24.2)
	Strongly disagree	48 (18.2)
**Competence in recommending contraceptive methods §**	Combined oral contraceptives	136 (51.5)
	Barrier methods	92 (34.8)
	Copper intrauterine device	44 (16.7)
	Levonorgestrel-releasing intrauterine device	36 (13.6)
	Emergency contraception	24 (9.1)
	Subdermal implant	20 (7.6)
	Progestin-only pills	16 (6.1)
	None	56 (21.2)

Domain B. Teratogenic Medication and Risk Assessment Practices

Variable	Category	n (%)
**Check contraceptive use before prescribing teratogenic medication**	Strongly agree	144 (54.5)

Variable	Category	n (%)
	Agree	72 (27.3)
	Neutral	28 (10.6)
	Disagree	8 (3.0)
	Strongly disagree	12 (4.5)
**Actions taken regarding contraception when prescribing potentially teratogenic medication §**	Verbal inquiry	60 (22.7)
	Written informed consent	40 (15.2)
	Referral to family planning services	40 (15.2)
	No action taken	204 (77.3)
**Evaluate drug–contraceptive interactions**	Strongly agree	72 (27.3)
	Agree	80 (30.3)
	Neutral	60 (22.7)
	Disagree	28 (10.6)
	Strongly disagree	24 (9.1)
**Reassess combined hormonal contraception during prolonged immobilisation**	Strongly agree	96 (36.4)
	Agree	80 (30.3)
	Neutral	32 (12.1)
	Disagree	32 (12.1)
	Strongly disagree	24 (9.1)

Domain C. Knowledge, Awareness, and Training

Variable	Category	n (%)
**Contraceptive choice may affect disease course‡**	Strongly agree	88 (33.8)
	Agree	144 (55.4)
	Neutral	24 (9.2)
	Disagree	4 (1.5)
	Strongly disagree	0 (0.0)

Variable	Category	n (%)
**WHO MEC awareness**	Never heard	200 (75.8)
	Heard but not familiar	56 (21.2)
	Know in detail	8 (3.0)
**WHO MEC mobile application awareness**	Yes	16 (6.1)
	No	248 (93.9)
**Postgraduate contraception training**	None	216 (81.8)
	Course	32 (12.1)
	Congress/meeting	8 (3.0)
	Online training	8 (3.0)
**Self-rated knowledge level (1–5)**	Very poor	44 (16.7)
	Poor	68 (25.8)
	Moderate	104 (39.4)
	Good	48 (18.2)
	Very good	0 (0.0)

Domain D. Perceived Barriers and System-Level Factors

Variable	Category	n (%)
**Perceived barriers §**	Topic outside my area	132 (50.0)
	Lack of up-to-date knowledge	128 (48.5)
	Time constraints	124 (47.0)
	System/organizational constraints	72 (27.3)
	Patients' lack of interest	60 (22.7)
**Ethical/legal responsibility when prescribing teratogenic drugs‡**	Yes	44 (16.9)
	Not sure	216 (83.1)
	No	0 (0.0)
**Referral to family planning clinic**	Never	152 (57.6)
	Rarely	4 (1.5)
	Often	8 (3.0)
	Always	100 (37.9)
**Perceived adequacy of family planning services**	Very adequate	4 (1.5)

Variable	Category	n (%)
	Adequate	24 (9.1)
	Partly adequate	44 (16.7)
	Inadequate	68 (25.8)
	No opinion	124 (47.0)
**Preferred training format** §	Online video	124 (47.0)
	Face-to-face seminar	64 (24.2)
	Brochure	44 (16.7)
	In-clinic case discussion	32 (12.1)
**Willingness to participate in free online training**	Yes	172 (65.2)
	No	8 (3.0)
	Undecided	84 (31.8)
**Most frequently used information source** §	Colleagues	136 (51.5)
	UpToDate	104 (39.4)
	Guidelines (WHO, CDC, etc.)	52 (19.7)
	Lecture notes	36 (13.6)
	None	44 (16.7)
**Top priority to improve services**	Provide physician training	108 (40.9)
	Increase FP unit capacity	64 (24.2)
	Develop clinical protocols	64 (24.2)
	Patient materials	28 (10.6)

Legend: Data are presented as *n* (%). Percentages were calculated using the number of available responses for each item. Unless otherwise indicated, the denominator was the total study sample (N = 264). § Multiple-response item; percentages may therefore sum to more than 100%. ‡ Four responses were missing for this item; percentages were calculated using

the available responses (N = 260). WHO MEC, World Health Organization Medical Eligibility Criteria for Contraceptive Use; FP, family planning; CDC, Centers for Disease Control and Prevention.
